# Supplementary material for: Association between Albumin Alterations and Renal Function in Patients with Type 2 Diabetes Mellitus
Source: Int J Mol Sci. 2024 Mar 9;25(6):3168. doi: 10.3390/ijms25063168 (PMC10970212; doi:10.3390/ijms25063168)
Supplement: Supplementary file 1 [file ijms-25-03168-s001.zip › ijms-2842343-supplementary.pdf]

SUPPLEMENTARY MATERIALS

Association between albumin alterations and renal function in patients with Type 2 Diabetes Mellitus

Marta Nugnes[1*], Maurizio Baldassarre[2,3*], Danilo Ribichini[4], Daniele Tedesco[1,5], Irene Capelli [3,6], Daniele Vetrano [3,6], Francesca Marchignoli [7], Lucia Brodosi [7], Enrico Pompili[2,3], Maria Letizia Petroni[7], Gaetano La Manna[3,6], Giulio Marchesini[7], [§] Marina Naldi[1,8], Manuela Bartolini[1]

[1]*Department of Pharmacy and Biotechnology, Alma Mater Studiorum University of Bologna, Italy.*

[2]*Unit of Semeiotics, liver and alcohol-related diseases, IRCCS Azienda Ospedaliero-Universitaria di Bologna, Bologna, Italy.*

[3]*Department of Medical and Surgical Sciences, Alma Mater Studiorum University of Bologna, Italy.*

[4]*Endocrinology and Diabetes Prevention and Care Department, IRCCS Azienda Ospedaliero-Universitaria di Bologna, Bologna, Italy*

[5]*Institute for Organic Synthesis and Photoreactivity, National Research Council, Bologna, Italy.*

[6]*Nephrology, Dialysis and Renal Transplant Unit, IRCCS Azienda Ospedaliero-Universitaria di Bologna, 40138 Bologna, Italy.*

[7]*Unit of Clinical Nutrition, IRCCS Azienda Ospedaliero-Universitaria di Bologna, Bologna, Italy.*

[8]*Centre for Applied Biomedical Research (CRBA), Alma Mater Studiorum University of Bologna, Italy.*

Supplementary Table S1. Anthropometric and clinical data of patients with DKD I-III.

	**T2DM+ DKD I**<br>n = 24	**T2DM+ DKD II**<br>n = 17	**T2DM+ DKD III**<br>n = 32	**p value**
**Anthropometric data**				
Age (years)	72 (68-73)	64 (61-74)	70 (68-74)	0.395
Male sex	17 (71)	11 (65)	24 (71)	0.895
BMI	32 (28-34)	31 (28-35)	33 (26-39)	0.787
**Drug therapy**				
Anti-hypertensives	21 (88)	15 (88)	26 (77)	0.434
ACE inhibitors	9 (38)	8 (47)	12 (35)	0.711
Angiotensin receptor blockers	9 (38)	6 (35)	12 (35)	0.983
Diuretics	6 (25)	8 (47)	18 (53)	0.097
Metformin	17 (71)	14 (82)	11 (32)	0.001
Insulin	7 (29)	11 (65)	22 (65)	0.016
Other Glucose-lowering drugs	19 (80)	13 (77)	11 (32)	<0.001
Sulfonylureas	5 (21)	3 (18)	2 (6)	0.215
DPP-4 inhibitors	6 (25)	4 (24)	3 (9)	0.206
GLP-1 Receptor agonists	7 (29)	7 (41)	7 (21)	0.300
SGLT-2 Inhibitors	3 (13)	0 (0)	0 (0)	0.036
Statin/fibrates	15 (63)	14 (82)	21 (62)	0.296
**Biochemical parameters**				
HbA1c (%)	7.1 (6.4-7.6)	7.1 (6.2-7.5)	7.1 (6.1-7.9)	0.920
Glucose (mg/dL)	121 (110-155)	123 (120-136)	124 (103-160)	0.922
Total cholesterol (mg/dL)	160 (140-177)	132 (123-142)	151 (128-159)	0.010
HDL (mg/dL)	45 (39-51)	35 (34-44)	38 (31- 42)	0.022
LDL (mg/dL)	78 (71-107)	70 (45-78)	84 (58-93)	0.073
Triglycerides (mg/dL)	173 (131-212)	158 (109-179)	155 (112-206)	0.645
Creatinine (mg/dL)	1.2 (1.0-1.4)	1.3 (1.2-1.4)	1.8 (1.5-3.5)	<0.001
UACR (mg/g)	13 (6-25)	78 (40-126)	312 (66-848)	<0.001
eGFR (mL/min/1.73m^2)	58 (52-59)	52 (46-55)	32 (23-39)	<0.001

Data is reported as mean and standard deviation, median and interquartile range or absolute number and frequencies.

ARBs: Angiotension-Receptor Blockers; ACE-Is: Angiotensin-Converting Enzyme Inhibitors
